# Responses of Bacterial Community Structure, Diversity, and Chemical Properties in the Rhizosphere Soil on Fruiting-Body Formation of *Suillus luteus*

**DOI:** 10.3390/microorganisms10102059

**Published:** 2022-10-18

**Authors:** Yixin Zhou, Zhichao Shi, Qiliang Pang, Xiufeng Liang, Hongtao Li, Xin Sui, Chongwei Li, Fuqiang Song

**Affiliations:** 1 Engineering Research Center of Agricultural Microbiology Technology, Ministry of Education, Heilongjiang University, Harbin 150500, China; 2 Heilongjiang Province Key Laboratory of Ecological Restoration and Resource Utilization for Cold Region, School of Life Sciences, Heilongjiang University, Harbin 150080, China; 3 Jiaxiang Research Academy of Industrial Technology, Jining 272400, China; 4 Heilongjiang Greater Hinggan Mountains Region Agriculture Forestry Research Institute, Gagdaqi 165100, China; 5 Swiss Federal Research Institute WSL, 8903 Birmensdorf, Switzerland

**Keywords:** mycorrhizal helper bacteria, rhizosphere bacteria, ectomycorrhizal fungus, fruiting-body formation, *Suillus luteus*

## Abstract

Mycorrhiza helper bacteria (MHB) play an important role in driving mycorrhizal formation. There are few reports on the relationship between bacteria and fruiting growths. Taking mycorrhizal rhizosphere soil from sporocarps of the *S. luteus* and non-mycorrhizal rhizosphere soil of the host plant (*Larix gmelinii*), we measured the bacterial community structure and diversity and chemical properties to clarify the effect of bacteria on fruiting-body formation. The bacterial diversity was significantly higher in mycorrhizal rhizosphere soil (*p* < 0.05) than that in non-mycorrhizal rhizosphere soil. The relative abundance of *Burkholderia*, *Bradyrhizobium*, *Pseudomonas*, and *Rhizobium* was significantly higher (*p* < 0.05) in mycorrhizal rhizosphere soil than in non-mycorrhizal rhizosphere soil. The soil organic matter (SOM), total nitrogen (TN), total phosphorus (TP), total potassium (TK), ammonium nitrogen (AN), available phosphorus (AP), available potassium (AK), and the activity of catalase, urease, and phosphatase in mycorrhizal rhizosphere soil were significantly higher (*p* < 0.05) than those in non-mycorrhizal rhizosphere soil. A redundancy analysis (RDA) showed that dominant bacteria are closely related to soil enzyme activity and physicochemical properties (*p* < 0.05). The boletus recruits a large number of bacteria around the plant roots that speed up nutrient transformation and increase the soil nutrient content, providing an important guarantee for mycelium culture and fruiting-body formation. These findings provide ideas for the nutritional supply of boletus sporocarps and lay the theoretical foundation for the efficient artificial cultivation of boletus.

## 1. Introduction

Mycorrhiza, a symbiotic association between higher plants and microorganisms, are commonly found in nature. In this symbiotic relationship, the host plant provides carbohydrates to mycorrhiza fungi through photosynthesis. In return, mycorrhiza fungi provide nutrients (e.g., phosphorus and nitrogen) to the host plant by absorbing mineral material from the soil [1]. Ectomycorrhizae (EM), arbuscular mycorrhizae (AM), orchid mycorrhizae (ORM), and ericoid mycorrhizae (ERM) are four main types of mycorrhizae with specific morphologies in plants and fungi [2]. Ectomycorrhizae are mainly found in the root systems of trees, and the fungal mycelium forms intertwined connectives or mycelial sheaths with the forest root system. Some of the mycelia can reach between the cortical cells of the host root system, forming interlinked Hartig’s net structures, but they do not invade inside the plant cells [3]. Ectomycorrhizae enhance plants’ ability to absorb water and mineral nutrients (e.g., phosphorus, potassium, nitrogen, and calcium) and provide metabolites such as auxin, vitamins, cytokinins, antibiotics, and aliphatic acids for plant growth. A study reported that large numbers of bacteria were living in the surroundings of some ectomycorrhizal fungi [4].

Both these bacteria and mycorrhizal fungi secrete enzymes into the soil, which are the main catalysts for organic matter decomposition, turnover, and mineralization and are the most active substances involved in ecosystem material cycling and energy flow [5,6,7]. Soil enzymes not only help plants adapt to changes in their environment but also improve the nutrients in the soil, which is important for plant growth [8].

Garbaye named these bacteria that can promote mycorrhizal growth, colonization, and the formation of symbiotic structures mycorrhization helper bacteria (MHB) [4,9]. Frey-Klett classified MHB based on their mode of function, stimulating the formation of mycorrhiza or affecting the functions of an already established symbiosis [10]. EM fungi and MHB are interdependent, with mycorrhizal fungi providing nutrients for the growth of bacteria through the release of secretions and bacteria supplying low-molecular-weight carbohydrates (e.g., alginate and polyols) for EM fungi [11,12]. Garbaye hypothesized that MHB regulates rhizosphere soil physicochemical properties to enhance mycorrhizal infestation and growth [9]. However, currently a lot of studies on the function of mycorrhiza helper bacteria focus on the promotion of mycorrhizal formation and fungal mycelial growth [10,13,14,15], but few studies focus on the relationship of ectomycorrhizal fungal and mycorrhiza helper bacteria. With this study, we would like to complement the interaction between bacteria and mycorrhizal fungi after the symbiosis formation. Therefore, this experiment took the *L. gmelinii* and *S. luteus* symbiosis system in the Daxinganling area as the research object. *S. luteus* is a common ectomycorrhizal mycorrhizal fungus of *L. gmelinii* in northeastern China. It can increase the diversity of rhizosphere microorganisms and improve the host’s resistance to pests and diseases [16,17]; moreover, it has economic value because it is tasty and nutritious [18]. By 2019, the demand for boletus in China reached 70.12 MT/a, with an average price of 100–180 CNY/kg (15–27 USD/kg), and the market sales exceeded CNY 12.632 billion (USD 1.89 billion). However, artificial cultivation for boletus is difficult and is still in the laboratory research stage. We aimed to determine (i) how bacterial communities trigger fruiting-body formation and (ii) which soil properties are the main determinants of bacterial diversity and community structure when sporocarps grow. Our experiment measured the bacterial community structure change and the physicochemical parameters of the mycorrhizal rhizosphere (MR) and non-mycorrhizal rhizosphere (NR) soil, explored the relationship between them, and revealed the nutrient requirements of *S. luteus*. At the same time, this study will provide an important theoretical basis for the artificial cultivation of boletus.

## 2. Materials and Methods

### 2.1. Sampling and Processing

**Figure 1:**
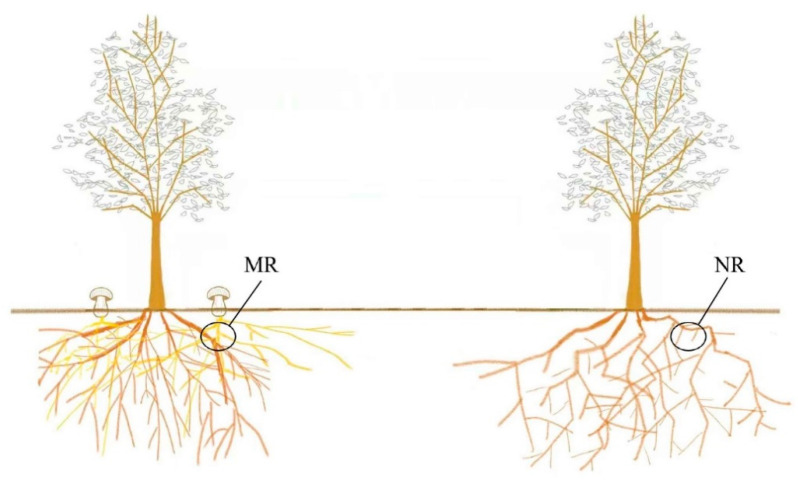
Schematic diagram of sample plot. “MR” refers to the mycorrhizal rhizosphere with a fruiting body. “NR” refers to the non-mycorrhizal rhizosphere without a fruiting body.

The samples were collected in September 2020 from Beishan Forest Park, Gagdachi District, Daxinganling Prefecture, Heilongjiang Province (50°43′50.63″ N, 124°13′49.78″ E), which is located in the remnants of the Daxinganling Mountains and is a typical low mountainous landscape with an average altitude of 472 m and a total area of 14,632.1 hm^2^. The forest cover is over 60%, dominated by *Larix gmelinii* as the climax community. This area is in the northern temperate zone with continental monsoon conditions. The average annual precipitation is 495 mm, and the average annual temperature is −1.2 °C. The soil is brown coniferous forest soil (in the genetic soil classification of China, GSCC). Mycorrhizal rhizosphere soil samples and non-mycorrhizal rhizosphere soil samples were collected from three *L. gmelinii* woodlots with *Suillus Luteus* sporocarps. For each wood plot, five soil samples were gently brushed and collected from mycelia 10–15 cm below the sporocarps or from the roots 10–15 cm below the *L. gmelinii* and then combined into one sample [19,20]. Three samples from mycelia were named MR1, MR2, and MR3; three samples from roots were named NR1, NR2, and NR3 (Figure 1).

The samples were collected, brought back to the local laboratory in Ziplock bags, and immediately sieved (pore size 2 mm). Each sample was divided into two parts; one was stored in a refrigerator at −80 °C for microbial community structure analysis, and the other was naturally air-dried for soil chemical property and enzyme activity determination.

### 2.2. DNA Extraction, PCR, and Sequencing

The total DNA of soil microorganisms was extracted using a Fast DNA SPIN Kit (MP Biomedicals, Irvine, CA, USA), and the concentration was detected by 1% agarose gel electrophoresis and Nanodrop 2000. The Power Clean DNA Clean-up Kit (MoBio, Carlsbad, CA, USA) was used for purity. The V3–V4 region of the bacterial 16S rRNA gene was amplified using universal primers 27F (5’-AGAGTTTGATCCTGGCTCAG-3’) and 1492R (5’- CTACGGCTACCTTGTTACGA-3’). The amplification conditions were: pre-denaturation at 98 °C for 1 min, denaturation for 10 s for 30 cycles, annealing at 50 °C for 30 s, extension at 72 °C for 30 s, and a final extension at 72 °C for 5 min. After PCR amplification, the detection was performed using the QuantiFluor™-ST Blue Fluorescence Quantification System (Promega). Sequencing libraries were prepared using an Illumina TruSeq Nano DNA LT Library Prep Kit, double-end sequencing was performed on the Miseq platform, and valid sequences were screened from the data based on barcode tag sequences and pre-primer sequences. The sequencing was performed by Shanghai Lingen Biotechnology Co., Ltd., (Shanghai, China).

### 2.3. Bioinformatic Processing

Sequences were processed and analyzed using UPARSE [21]. Non-repetitive sequences were extracted from the optimized sequences after removing single sequences without repeats. Operational taxonomic units (OTU) were clustered sequences without non-repetitive or chimeras according to 98.65% similarity using USEARCH v10 (http://drive5.com/usearch accessed on 31 March 2021). All sequences were normalized. To obtain taxonomic information on the species corresponding to each OTU, the uclust algorithm (v1.2.22q) was used to taxonomically analyze the OTU representative sequences and count each sample’s community composition at each taxonomic level. The 16s bacterial ribosome database (Silva Release 138.1, http://www.arb-silva.de accessed on 31 March 2021) was used to annotate the taxonomy of each sample.

### 2.4. Chemical Properties

Soil chemical properties include organic matter (SOM), total nitrogen (TN), total phosphorus (TP), total potassium (TK), ammonium nitrogen (AN), available phosphorus (AP), available potassium (AK), and soil pH, reflecting the comprehensive characteristics of soil nutrition. Soil organic matter, ammonium nitrogen, available phosphorus, available potassium, and pH were determined using a soil nutrient tester (TPY-8A, Zhejiang Top, Hangzhou, China). Soil total phosphorus was assayed using a continuous flow analytical system (SKALAR SAN++, Breda, The Netherlands). Soil total potassium was quantified using inductively coupled plasma-atomic emission spectrometry (ICPS-7500, Shimadzu, Japan). Soil total nitrogen was determined using an elemental analyzer (VarioEL III, Langenselbold, Germany). Three biological replicates per sample were tested [22,23].

### 2.5. Enzymic Activity

The activity of catalase was determined by permanganate titration. The urease was determined by the sodium phenol–sodium hypochlorite colorimetric method, and phosphatase was determined by the sodium phenyl phosphate colorimetric method according to the method of reference [24]. Three biological replicates per sample were tested.

### 2.6. Statistical Analysis

A one-way ANOVA was performed using SPSS 21.0 software to characterize significant differences at a threshold value of *p* < 0.05. Using R (version 3.6.3), a Venn diagram, relative abundance diagram, and cluster analysis diagram were created by “ggplot2”. The α-diversity index was calculated using Mothur v.1.35.1 (http://www.mothur.org accessed on 31 March 2021) [25]. A bacterial variance analysis was performed using STAMP v2.1.3 software [26]. A redundancy analysis (RDA) was conducted to identify the correlations between soil physicochemical parameters, enzymes, and dominant bacteria using Canoco 5 software.

## 3. Results

### 3.1. Fruiting Body and Mycorrhizal Morphology

**Figure 2:**
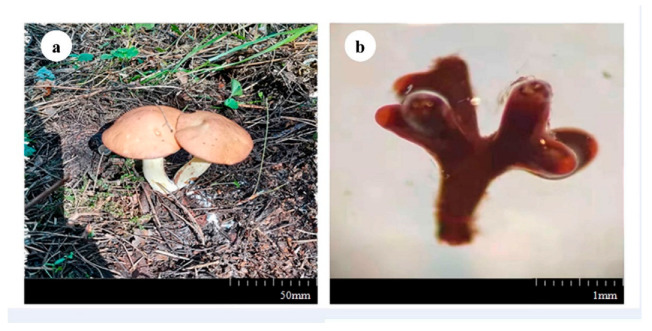
(**a**) Morphosis of the fruiting body of *Suillus luteus*. (**b**) Morphosis of the mycorrhizae of *Suillus luteus* (10×).

The cap was 4–8 cm in diameter, hemispherical, light brown or reddish-brown, with a smooth or sticky surface. The gills were pale white or yellowish. The length of the stipe was 3.4–9.4 cm, and the diameter was 0.8–2.5 cm, columnar, waxy yellow, with scattered small glandular dots on the surface. The ring was in the upper part of the stalk, thin-film-like, and brown (Figure 2a). *S. luteus* forms coral-like mycorrhizae with the larch root system. The length of the mycorrhizae was about 1 mm, and they were dark brown (Figure 2b).

### 3.2. Sequence Data Processing

**Figure 3:**
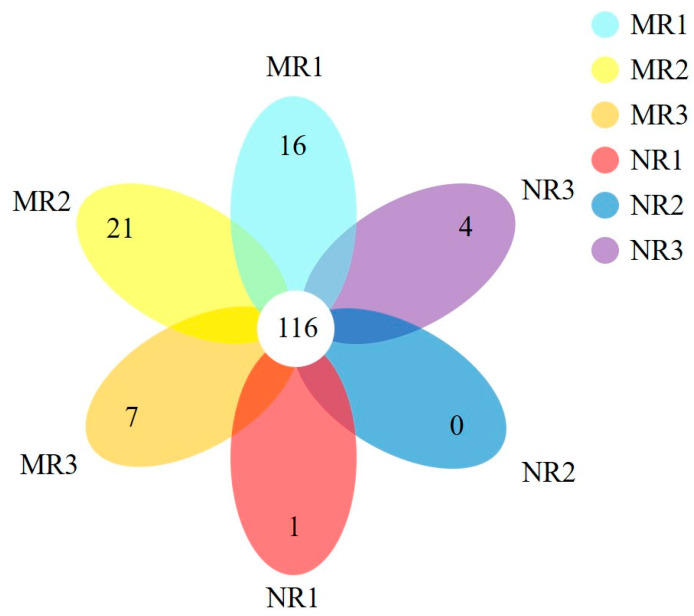
Venn diagram of bacteria. Each color in the Venn diagram represents a sample. Overlapping circles represent the number of OTUs shared by the sample; non-overlapping sections represent the number of OTUs specific to the sample.

Using PacBio full-length 16s high-throughput sequencing, 60508 sequences were obtained after quality control. After a similarity analysis, >98.65% of the sequences were classified into the same out, and there was a total of 289 OTUs. OTUs with >1% abundance were screened for analysis, and the results are shown in Figure 3.

The two soil types contained 116 dominant OTUs, accounting for 40.14% of the total number of OTUs (Figure 3). This indicated the bacterial microbial community structure was relatively similar in both MR and NR. A total of 16 OTUs were endemic to MR1, 21 OTUs were endemic to MR2, and 7 OTUs were endemic to MR3. Only one endemic OTU was in NR1, while there were no endemic OTUs in NR2, and four endemic OTUs were in NR3. This showed that there were significantly more bacterial species in MR than in NR (*p* < 0.05).

The Shannon and ACE indices of MR were significantly higher than those of NR (*p* < 0.05), and the Simpson index of MR was significantly lower than that of NR (Table 1, *p* < 0.05). Therefore, the community diversity of MR was significantly higher than that of NR.

### 3.3. Clustering and Variation Analysis of Bacterial Communities

The dominant phyla (>1%) in MR were Proteobacteria (53.18 ± 7.48%), Acidobacteria (29.60 ± 4.85%), Actinobacteria (4.08 ± 1.42%), Verrucomicrobia (2.62 ± 1.28%), and Gemmatimonadota (2.61 ± 1.56%). The dominant phyla (>1%) in NR were Proteobacteria (36.46 ± 5.63%), Acidobacteria (32.5 ± 5.35%), Actinobacteria (8.24 ± 2.44%), Verrucomicrobia (8.64 ± 3.75%), and Gemmatimonadota (8.25 ± 1.58%). Proteobacteria and Actinobacteria accounted for a greater proportion of both soils, and the relative abundance of Proteobacteria in MR was significantly higher than in NR (*p* < 0.05).

**Figure 4:**
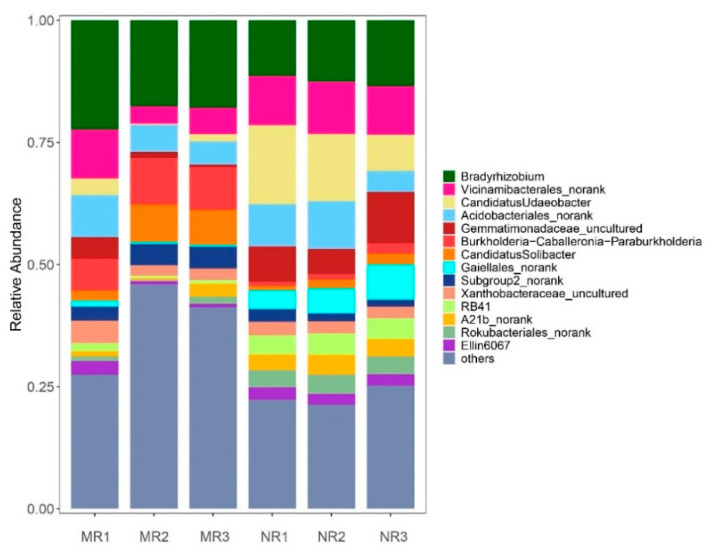
Relative abundance of dominant bacteria genera in rhizosphere soil. The community component of the dominant bacteria in the soil of genera that represented >1% in at least one sample, annotated by the Silva Release 138.1 database.

At the genus level, the dominant genera (>1%) [27] in MR soil included *Bradyrhizobium* (19.30 ± 2.17%), *Burkholderia* (8.26 ± 1.26%), *Vicinamibacterales*_*norank* (6.32 ± 2.71%), *Acidobacteriales*_*norank* (6.28 ± 1.67%), *Candidatus*_*Solibacter* (5.58 ± 2.54%), *Subgroup* 2_*norank* (3.88 ± 0.74%), *Xanthobacteraceae*_*norank uncultured* (3.03 ± 1.11%), *Granulicella* (2.82 ± 1.65%), and *Gemmatimonas* (2.10 ± 1.68%). The dominant genera in NR soil included *Candidatus Udaeobacter* (12.47 ± 3.68%), *Bradyrhizobium* (12.45 ± 0.85%), *Vicinamibacterales_norank* (10.23 ± 0.35%), *Acidobacteriales_norank* (7.54 ±2.36%), *Gaiellales_norank* (5.43 ± 1.39%), *Gaiella* (4.23 ± 0.18%), *Rokubacteriales_norank* (3.72 ± 0.17%), *A21b_norank* (3.56 ± 0.37%), and *Ellin6067* (2.39 ± 0.13%) (Figure 4).

**Figure 5:**
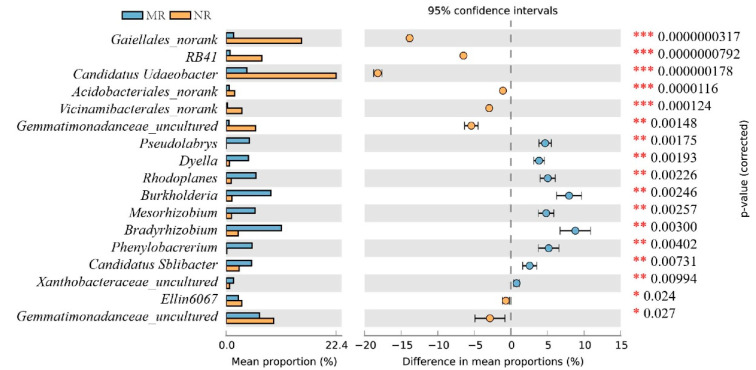
Analysis of differences in the abundance of soil bacterial genera levels. Differences in bacterial abundance between MR and NR. In the figure, “*” indicates a significant difference between the two data sets (*n* = 3, *p* < 0.05); “**” indicates a highly significant difference between the two data sets (*n* = 3, *p* < 0.01); “*** “ indicates a highly significant difference between the two data sets (*n* = 3, *p* < 0.001) using Welch’s *t*-test.

The differences in the dominant bacterial genera in the two soils were analyzed (Figure 5). The differential bacteria were divided into 20 genera. In MR soil, *Bradyrhizobium* and *Burkholderia* were significantly higher (*p* < 0.05) than those in NR soils, and *Candidatus Udaeobacter* and *Vicinamibacterales_norank* were significantly lower (*p* < 0.05) than those in NR. In addition, *Caulobacteraceae_uncultured*, *Kibdelosporangium*, *Solirubrobacteraceae*_*uncultured*, and *Blastocatellaceae_uncultured* were found to be endemic and relatively abundant in MR soils. Thus, the bacterial diversity of the MR soil was richer, and the relative abundance of several important soil bacteria, such as *Rhizobium* and *Bradyrhizobium*, was higher than in NR.

### 3.4. Physicochemical Characteristics of Soil

The concentrations of SOM, TN, TP, TK, AN, AP, and AK in MR were all significantly higher than those in NR (*p* < 0.05), and the concentration of H^+^ increased in soil with mycelia or fruiting bodies, leading to a decrease in the soil pH. As shown, the MR soil had higher nutrients than the NR soil.

**Figure 6:**
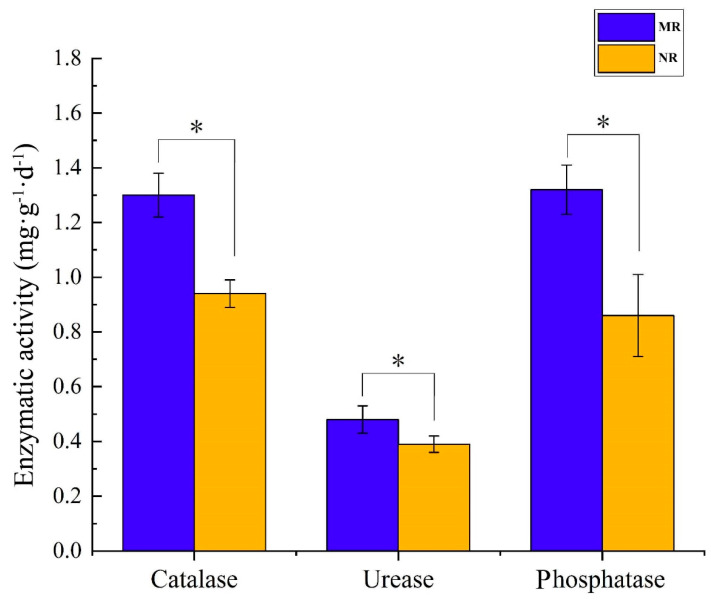
The activities of catalase, urease, and phosphatase in the mycorrhizal rhizosphere of *S. luteus* and non-mycorrhizal rhizosphere. “*” indicates significant differences (*n* = 3, *p* < 0.05).

The activities of three soil enzymes are shown in Figure 6. Compared to the enzyme activities in NR, the activities of catalase, urease, and phosphatase in MR were significantly higher (*p* < 0.05). While catalase increased by 38.3%, urease activity increased by 23.1% and phosphatase activity increased by 53.49%.

**Figure 7:**
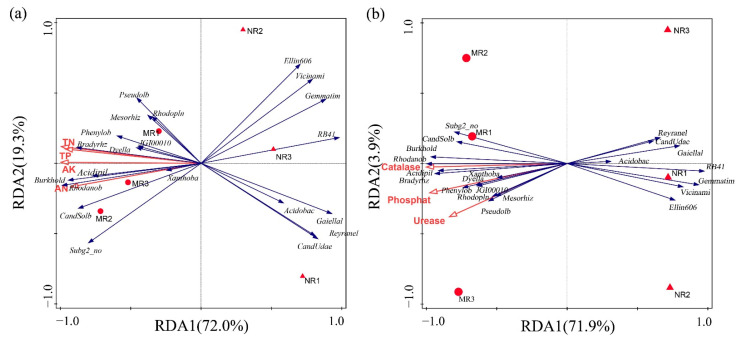
(**a**) Redundancy analysis among dominant bacterial genera (>1%) and soil physicochemical parameters. Red arrows represent environmental factors, and blue arrows represent bacterial genera. (**b**) Redundancy analysis of dominant bacterial genera and soil enzyme activity. The red arrows represent soil enzyme activities, and the blue arrows represent bacterial genera. The positions and lengths of the arrows represent the direction and intensity of the influence of indicators on the microbial community, as validated by Monte-Carlo permutation tests.

The community structures within the MR and NR groups were similar, but there were significant differences between the groups in terms of community structure and physicochemical parameters (Figure 7).

AK, TN, TK, and TP were positively correlated with the relative abundance of *Bradyrhizobium*, *Burkholderia*, *Acidobacteriales*, *Candidatus Rhodanobacter*, and *Phenylobacterium* and negatively correlated with *Vicinamibacterales*, *Gemmatimonadaceae*, *RB41*, *Gaiellales*, and *Acidipila-Silvibacterium* (Figure 7a, *p* < 0.05). AK was the most important physicochemical indicator affecting the relative abundance of bacteria. The activities of catalase, urease, and phosphatase were positively correlated with the relative abundance of *Phenylobacterium*, *Dyella*, *Rhodoplanes*, *Bradyrhizobium*, *Rhodanobacter,* and *Burkholderia* and were negatively correlated with *Gemmatimonadaceae*, *Vicinamibacterales*, *Acidobacteriales*, *Gaiellales,* and *Reyranella* (Figure 7b, *p* < 0.05). The dominant bacteria in mycorrhizal rhizosphere soil were more closely related to soil enzyme activity and physicochemical properties.

## 4. Discussion

### 4.1. The Relative Abundance of Bacteria in the Mycorrhizal Rhizosphere Was Significantly Higher Than in the Non-Mycorrhizal Rhizosphere

Mycorrhization helper bacteria (MHB) are widespread around the roots of different plant–fungal mycorrhizae. Past literature has suggested that *Agrobacterium* [28], *Burkholderia* [29], *Pseudomonas* [30], *Bacillus* [31], *Paenibacillus* [32], and the actinomycete *Streptomyces* [33] are mycorrhization helper bacteria. In our findings, the mycorrhizal rhizosphere exhibited a lower Simpson index and higher Shannon and ACE indices (Table 1), which indicated that this area possessed higher soil bacterial diversity and evenness. At the phylum level, the bacteria in the two soils consisted mainly of the phyla Proteobacteria, Acidobacteriota, Actinobacteriota, Verrucomicrobiota, Gemmatimonadota, Methylomirabilota, Bacteroidota, Myxococcota, Planctomycetota, and Chloroflexi. Among these bacteria, many have been identified by both culture-dependent and culture-independent methods as belonging to our specific taxa, including phyla such as α-, β-, and γ-Proteobacteria and Actinobacteria, more specifically, Bacillales, Burkholderiales, Actinomycetales, Rhizobiales, and Pseudomonadales [34]. In Figure 4, at the genus level, *Burkholderia* and *Pseudomonas* are proven to be MHB, while *Bradyrhizobium* and *Rhizobium* have been documented to be associated with promoting the growth of mycorrhizal fungi [35,36]. The relative abundance of these four bacteria was significantly higher in mycorrhizal rhizosphere soil than in non-mycorrhizal rhizosphere soil (Figure 5, *p* < 0.05). The nutritional requirements of bacteria and fungi play a role in the interaction. The mycorrhizal fungi *Laccaria bicolor* synthesize trehalose, which stimulates the growth and chemotaxis of the MHB *Psuedomonas fluorescens* and acquires thiamine from *p. flourescens* [37]. Furthermore, MHB have the ability to receive nourishment from the outer hyaline spore layer, and these bacteria were found to possess the ability to produce cell-wall-degrading enzymes, including cellulase, chitinase, and protease, and are capable of breaking down exopolysaccharides, which may lead to their increase in relative abondance [38]. In addition, several bacteria were endemic and relatively abundant in the mycorrhizal rhizosphere, including *Caulobacteraceae*, *Kibdelosporangium*, *Solirubrobacteraceae*, *Blastocatellaceae*, and *Blastocatellaceae*, of which *Kibdelosporangium* is an actinomycete and can produce antibacterial and nematicidal active substances [39], safeguarding the health of the soil environment and thus promoting the growth of host plants [40,41]. Due to the selective specificity of MHB with rhizosphere symbiotic fungi, an increase in the relative abundance of MHB in the mycorrhizal root interiors provides an external environment for mycelial growth and the mycorrhizal colonization of boletus, similar to the findings of Wu et al. [42].

### 4.2. MHB in Mycorrhizal Rhizosphere Soils Promotes Nutrient Transformation and Indirectly Provides Nutrient Reserves for Sporocarp Formation

In natural ecosystems, nitrogen is an important nutrient for plant growth, and an important function of mycorrhizal fungi is to provide nitrogen and phosphorus to the host plant. Govindarajulu et al. [43] and Jin et al. [44] demonstrated in site-controlled experiments that mycorrhizal fungi contribute up to 30% and 50% of the nitrogen to the host plant. Rhizosphere bacteria assist mycorrhizal roots in nutrient metabolism by producing urease and phosphatase enzymes that activate the conversion of soil nitrogen and phosphorus into ammoniacal nitrogen (NH_4_^+^-N), nitrate nitrogen (NO_3_^−^-N), and phosphate (PO_4_^3−^) that can be directly absorbed and used by plants [27,45,46,47]. In our study, the composition of the bacterial communities was significantly correlated with total N, total P, available K, and ammonium N (Table 2; Figure 7a). The relative abundance of *Rhizobium* and *Bradyrhizobium* in mycorrhizal rhizosphere soil was significantly higher (*p* < 0.05) than in non-mycorrhizal rhizosphere soil (Figure 5), and both genera of bacteria had nitrogen fixation functions, increasing the nitrogen content of mycorrhizal rhizosphere soil and providing nutrients for the growth of mycorrhizae [48]. At the same time, MHB enhance effective phosphorus absorption and utilization by secreting extracellular phosphatase to degrade organic phosphorus [49,50,51]. Mycorrhizal fungi secrete various metabolites (mainly carbohydrates, organic acids, and amino acids) into the rhizosphere, recruiting functional bacteria, including phosphate-solubilizing bacteria and potassium-solubilizing bacteria, to colonize the mycelial surface and secrete soil enzymes such as phosphatase, urease, and catalase, thereby increasing the soil enzyme activity and the nutrients in the rhizosphere [52], which is consistent with the findings that the mycorrhizal rhizosphere soil enzyme activity and nutrient contents were significantly higher than those of the non-mycorrhizal rhizosphere.

It can be seen from Figure 6 that all three soil enzyme activities of mycorrhizal rhizosphere soils were higher than those of non-mycorrhizal rhizosphere soils (*p* < 0.05). The contents of total P and available P in mycorrhizal rhizosphere soils were significantly higher than those of non-mycorrhizal rhizosphere soils (*p* < 0.05), consistent with the variation pattern of phosphatase enzyme activities; the contents of total N and ammonium nitrogen were significantly higher than those of non-mycorrhizal rhizosphere soils (*p* < 0.05), consistent with the variation pattern of phosphatase enzyme activities. The organic matter was significantly higher than that of non-mycorrhizal rhizosphere soil (*p* < 0.05), consistent with the change in catalase enzyme activity (Table 2; Figure 7b), indicating that, due to the increase in enzyme activity of mycorrhizal rhizosphere soil, the process of material cycling and energy conversion in the soil was active. These soil enzymes played the same role as in previous studies to accelerate the conversion rates of carbon, nitrogen, and phosphorus in the soil and accumulated nutrients in the mycorrhizal rhizosphere [53,54,55], providing nutrients and energy reserves for the growth of mycelia and sporocarps of boletus [56].

## 5. Conclusions

Through the study of the bacterial community structure and physicochemical indicators in the mycorrhizal rhizosphere soil of *S. luteus*, we found that in the mycorrhizal rhizosphere of the host plant the metabolic activity of mycorrhizal fungi was enhanced due to an increase in the relative abundance of mycorrhizal helper bacteria, and they were recruited with secreted metabolites. Mycorrhizal helper bacteria are mostly functional bacteria that significantly increase the enzymatic activity of rhizosphere soil enzymes, speed up nutrient transformation, and increase soil nutrient content, providing an important guarantee for *S. luteus* mycelium culture and fruiting-body formation. The findings clarify the relationship between mycorrhizal fungi and rhizosphere microorganisms, as well as soil enzymatic activity and physicochemical indicators, and provide an important theoretical basis for the realization of the artificial cultivation of boletus.

## Figures and Tables

**Table 1:** Soil bacterial diversity index.

Sample	Chao1 Index	Shannon Index	Simpson Index	ACE Index	Coverage
MR	208.77 ± 6.41 a	4.61 ± 0.07 a	0.0165 ± 0.0006 b	205.81 ± 6.38 a	0.9947 ± 0.0022 a
NR	200.25 ± 7.67 a	4.50 ± 0.04 b	0.0235 ± 0.0035 a	193.97 ± 2.37 b	0.9958 ± 0.0009 a

Note: Data in the table are means ± standard error. Different letters in the same column indicate a significant difference among the mean values (*p* < 0.05), as calculated by Mothur.

**Table 2:** The chemical properties of different soil samples.

Sample	pH	SOM (g/kg)	TN (g/kg)	TP (g/kg)
MR	5.77 ± 0.026 a	51.93 ± 2.26 a	3.96 ± 0.35 a	0.65 ± 0.04 a
NR	5.83 ± 0.012 b	45.27 ± 1.96 b	2.80 ± 0.62 b	0.52 ± 0.06 b
	**TK (g/kg)**	**AN (mg/kg)**	**AP (mg/kg)**	**AK (mg/kg)**
MR	3.72 ± 0.17 a	57.92 ± 2.27 a	16.47 ± 1.25 a	244.67 ± 22.37 a
NR	3.35 ± 0.10 b	47.77 ± 1.71 b	13.87 ± 1.19 b	185.33 ± 20.31 b

Note: Data in the table are means ± standard error. Different letters in the same column indicate a significant difference among the mean values (*p* < 0.05) based on the least significant difference. SOM, soil organic matter; TN, total nitrogen; TP, total phosphorus; TK, total potassium; AN, available nitrogen; AP, available phosphorous; AK, available potassium. Three biological replicates were tested for each sample.

## Data Availability

Data were deposited into the figshare, and the DOI is https://doi.org/10.6084/m9.figshare.21345879.
